# Assessment of uterine caruncles, uterine cervix, and vulva during the postpartum period in Kivircik ewes with shear-wave elastography

**DOI:** 10.3389/fvets.2024.1484189

**Published:** 2024-12-31

**Authors:** Zeynep Günay Uçmak, İbrahim Kurban, Melih Uçmak, Mehmet Fatih Özbezek, Mehmet Ragıp Kılıçarslan, Sokol Duro, Tomasz Szara, Ozan Gündemir

**Affiliations:** ^1^Obstetrics and Gynaecology Department, Faculty of Veterinary Medicine, Istanbul University-Cerrahpaşa, Istanbul, Türkiye; ^2^Equine and Training Program, Vocational School of Veterinary Medicine, Istanbul University-Cerrahpaşa, Istanbul, Türkiye; ^3^Institute of Graduate Studies, Istanbul University-Cerrahpaşa, Istanbul, Türkiye; ^4^Department of Anatomy, Faculty of Veterinary Medicine, Agricultural University of Tirana, Tirana, Albania; ^5^Department of Morphological Sciences, Institute of Veterinary Medicine, Warsaw University of Life Sciences-SGGW, Warsaw, Poland; ^6^Department of Anatomy, Faculty of Veterinary Medicine, Istanbul University-Cerrahpasa, Istanbul, Türkiye

**Keywords:** caruncula, cervix, elastography, postpartum period, vulva

## Abstract

**Introduction:**

This study aims to quantify the shear wave speed (SWS) and stiffness of the uterine cervix (close to the internal cervical ostium (IOC) which is the cranial portion of cervix and close to the external cervical ostium (EOC) which is the caudal portion of cervix), caruncular areas, and vulvar labia during the postpartum period in healthy Kivircik ewes by using shear-wave elastography. Power Doppler ultrasonography was performed to evaluate the color pixel percentage (CPP) of the caruncles.

**Methods:**

The study included 13 healthy pregnant Kivircik ewes, which were randomly selected. A total of 12 measurements were taken from the uterine cervix and vulva from the postpartum first day to PP42 (daily for the first week and weekly from PP14 to PP42). However, only eight measurements were obtained from the caruncles because they could not be visible after day 14.

**Results:**

The time-dependent differences in the widest cross-sectional diameter of the caruncles were statistically significant (*p* < 0.001) both in ewes giving birth to singletons and twins. As a result of power Doppler ultrasonography examination, the time-dependent differences in the CPP of the caruncles were statistically significant (*p* < 0.01) in ewes giving birth to both singletons and twins. The diameter of the cervix at PP3 was significantly higher than the ones at PP14, PP21, PP28 (*p* < 0.05). The SWS and stiffness in the IOC for all ewes at PP35 were significantly higher than the ones at PP1, PP4, PP7, and PP14 (*p* < 0.05 and *p* < 0.01; respectively). However, the time-dependent differences in SWS and stiffness in the EOC were not statistically significant (*p* > 0.05). The time-dependent differences in the SWS and stiffness in the vulva were statistically significant (*p* < 0.001) in ewes giving birth to both singletons and twins.

**Discussion:**

In conclusion, it is possible to describe the changes throughout the postpartum period and evaluate the involution of the uterine cervix, caruncles, and vulvar labia and tissue stiffness with significant results by B mode ultrasonography, power Doppler and shear wave elastography. We provided valuable information to elucidate the differences in the involution process of the uterine cervix, caruncles, and vulva concerning the number of offspring during the postpartum period in Kivircik ewes.

## Introduction

The puerperal and postpartum periods are characterized by many physiological changes in the genital organs after delivery in ewes (1). Uterine involution is defined as the return of the uterus to its non-pregnant status and physiological function after parturition (2). Involution of the uterus is remarkably high in the first week of the postpartum period but is influenced by breed, management, season, dystocia, and suckling (3, 4). The completion period of uterine involution was shorter for ewes that lambed at the end of winter than for those lambing at the onset of summer (5). Also, the parity affected uterine involution, completed earlier in primiparous than in pluriparous ewes (6). The rate of uterine involution in ewes with singleton parturition was higher than that with triplet parturition. Thus, different litter sizes significantly affected postpartum uterine horn recovery more than the caruncles (2). Complete uterine involution occurs between 17–40 days of postpartum (7), and is determined by the size of the uterus and the restoration of the endometrium (8). Uterine involution in ewes involves the remodeling of both caruncular and intercaruncular areas of the uterine wall and the termination of differentiated uterine gland functions characteristic of pregnancy (9). After the expulsion of the placenta, the caruncles assume a concave shape, and their morphological appearance changes from concave to convex in the regression phase (7). Complete involution of caruncles takes 28 days, while complete regeneration takes more than 4 weeks after parturition (10, 11). It involves necrosis and separation of septa from underlying stroma and regrowth of epithelium from the caruncular edges (10). Due to the similar echotexture of the caruncles and the endometrium, it can be challenging to differentiate caruncles from endometrium foldings using ultrasonography around days 5–8 (7). The contour of the caruncle may be visible as round or oval from days 3 to 10 postpartum, and its diameter is undetectable after day 10 (2).

Diagnostic ultrasound has become an essential tool in veterinary medicine and provides an inexpensive, non-invasive method for further examination of the reproductive tract in male and female farm sheep (12, 13). Doppler ultrasonography provides information about blood flow and vascular perfusion. There are various reports on changes in uterine blood flow during the postpartum period in Kivircik sheep (14, 15). Ultrasonographic elastography enables the assessment of tissue elasticity and stiffness, and qualitative and quantitative applications of elastography are widely used in various reproductive organs in ewes (1, 16, 17). Maternal and fetal tissue elasticity in pregnant ewes was quantified by elastographic analysis, and the shear wave velocity of placentoma remained constant throughout gestation (17). Peralta et al. (16) quantify cervical stiffness using shear wave elastography in preterm birth and induced labor in pregnant sheep. Mariano et al. (1) evaluated the stiffness of the uterine wall (endometrium/myometrium) during the involution in healthy Santa Inês ewes by using Acoustic Radiation Force Impulse (ARFI) elastography.

This study aims to quantify the shear wave speed (SWS) and stiffness of the uterine cervix (close to the internal cervical ostium (IOC) which is the cranial portion of cervix and close to the external cervical ostium (EOC) which is the caudal portion of cervix), caruncular areas, and vulvar labia during the postpartum period in healthy Kivircik ewes by using shear-wave elastography. Also, power Doppler ultrasonography was performed to evaluate the color pixel percentage (CPP) of the caruncles.

## Materials and methods

This study was approved by the Unit Ethics Committee of İstanbul University-Cerrahpaşa, Faculty of Veterinary Medicine, İstanbul, Türkiye (Approval No. 2023/22).

### Animals and study design

The study was conducted at the Istanbul University-Cerrahpaşa Faculty of Veterinary Medicine Sheep Farm located at 40°59′19.3″N, 28°43′36.1″E during the parturition season (March to May). Thirteen healthy pregnant Kivircik ewes were randomly selected [body weight: 55–60 kg, body condition score (BCS): 3.5/5]. A semi-intensive feeding program was implemented on ewes between the ages of 2–4 years. The ultrasonography measurements began to be obtained 24 h after the delivery. The first measurement taken 24 h after birth was called postpartum 1 (PP1). Ultrasound measurements were taken daily for a week (PP1 to PP7) and weekly from the first week until the 42nd postpartum day (PP14, PP21, PP28, PP35, PP42). All measurements were performed by the same operator and at the same period of the day (9:00–11:00 a.m.).

### Ultrasound examinations

**Figure 1:**
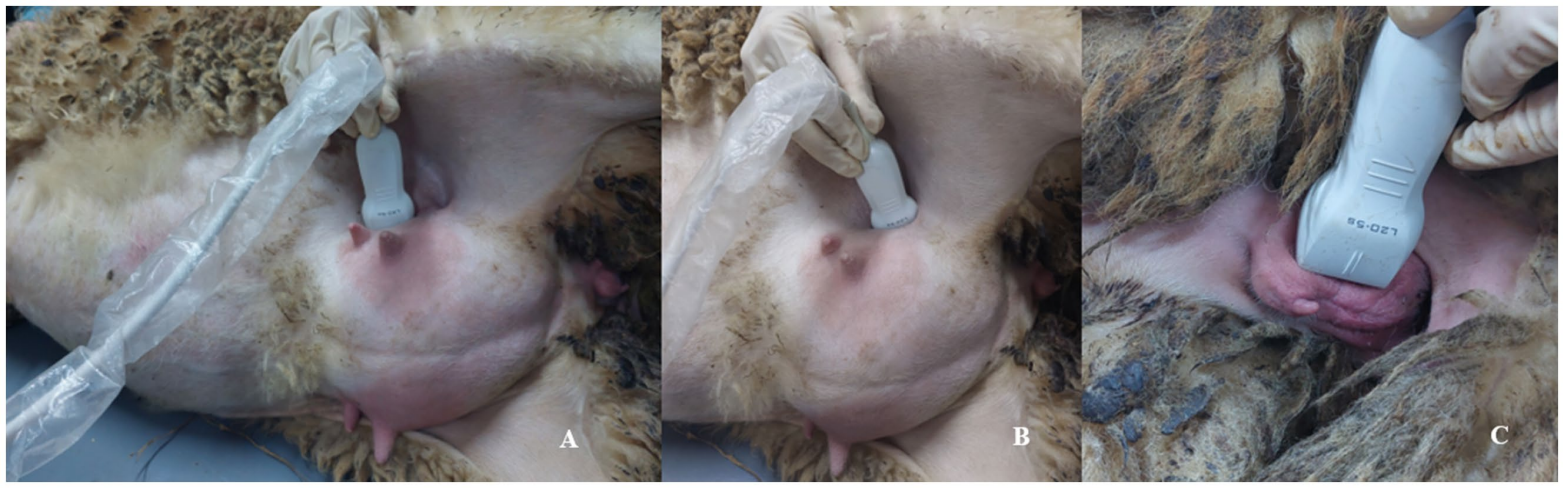
Posture of the sheep and positioning of the transducer during an ultrasound examination. **(A)** Evaluation of the uterine caruncles, transabdominally. **(B)** Visualisation of the cervix in the proximal lateral side of the mammary lobes. **(C)** Evaluation of the vulva with vertically positioned transducer.

All ultrasound measurements (B-mod, power Doppler ultrasound, shear-wave elastography) were performed transabdominally by using Resona i9 (Mindray, China) with linear transducer (L20-5 s). All ewes were laid on their right side (Figure 1A). The uterine caruncles were evaluated transabdominally. Also, the uterine cervix was visualized in the proximal lateral side of the mammary lobes in a longitudinal view (Figure 1B). For evaluation of the vulva, the transducer was positioned vertically on both vulvar labia (Figure 1C).

### B-mod ultrasonography

The widest cross-sectional diameters of uterine caruncles and uterine cervix were measured by B-mode ultrasonography. Additionally, the mean thickness of both vulvar labia was recorded. The settings of the B-mod ultrasound examinations in the caruncles, cervix, and vulva were as follows: the frequency (harmonic) (FH) was 18, gain (G) was 55, iBeam was +2, iclear was +3, dynamic rate (DR) was 130, SSI was 1,540.

### Power Doppler ultrasonography

Power Doppler images were quantified by measuring the color pixel percentage (CPP). The calculations were performed automatically by the ultrasound device. The vascularization in caruncles was evaluated in this study. The settings of the power Doppler ultrasonography were frequency (F) 11, G 60, pulse repetition frequency (PRF) 1.5 k, and Wall filter (WF) 215 Hz. Before elastography examinations, power Doppler was performed to visualize the blood vessels in the caruncles and cervix.

### Shear wave elastography

All measurements were performed in avascular areas of the relevant tissues (uterine caruncles, cervix, and vulva). The settings during elastography examination were HQE off, quality penetration Estory frequency (Q Pen), Map E2, and opacity (OP) was 4, iLay was off, and filter was 1 for all evaluated tissues. Stiffness (EkPa) and SWS (Cs, m/sn) were measured in the caruncle, cervix, and vulva. Qualitative and quantitative image analyses were activated. During elastography measurements, the motion stability index (M-STB index) was defined as green with ≥4 stars, and the reliability index (RI) was ≥85%. For quantification of the elasticity, the region of interest (ROI) was circular and kept constant at 2 mm in diameter for each tissue. The mean value of 4 ROI measurements was taken to determine the elasticity of the caruncles and vulva.

A total of 4 ROI areas which 2 ROIs close to the internal cervical ostium (IOC) where is the cranial portion of cervix and 2 ROIs close to the external cervical ostium (EOC) where is the caudal portion of cervix, were taken to evaluate the elasticity of the uterine cervix in a longitudinal view.

### Statistical analysis

Statistical analysis was performed by using the SPSS 23.0 program. The normal distribution of the data was checked with the Shapiro–Wilk test. The homogenicity of variance was analyzed using Levene’s test. The differences between ewes that gave birth to single offspring and those that gave birth to twins were evaluated using the t-test. The time-dependent differences in caruncles, cervix, and vulva were assessed using the General Linear Model and Repeated Measures of Anova. Tukey test was performed to evaluate the differences in the parameters between the examination days. Associations of the evaluated parameters (thickness/diameter, CPP%, SWS-m/sn, stiffness-EkPA) in related tissues were assessed with Pearson’s correlation test. Results were expressed as the means and standard errors. Significance was accepted as *p* < 0.05.

## Results

All ewes expulsed their placenta within 9 h after the delivery. Four of the 13 ewes had twin offsprings, while the rest of the nine ewes had singleton offsprings. A total of 12 measurements were taken on day PP1 to PP7-PP14-PP21-PP28-PP35-PP42 from the uterine cervix and vulva. Only eight measurements were obtained from uterine caruncles because they could not be visible after the day PP14.

### Uterine caruncles

**Figure 2:**
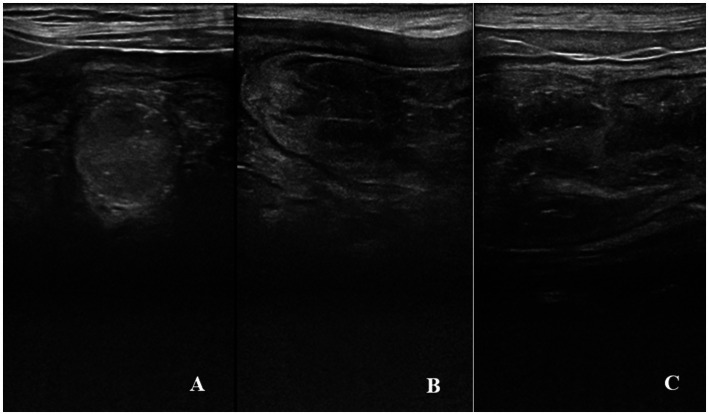
B-mod ultrasonographic evaluation of uterine caruncles. **(A)** Spherical shape of a caruncle at PP1. **(B)** Caruncular area with an inconsistent and blurred appearance at PP7. **(C)** Dispersed caruncular area at PP14.

The time-dependent differences in the broadest cross-sectional diameter of the caruncles were statistically significant in ewes giving birth to both singletons (*p* < 0.001) and twins (*p* < 0.01) (Table 1). A decrease in the diameter of the caruncles was determined from PP1 to PP14. While the caruncular diameter of all ewes at PP1 was not different from at PP2, it was significantly higher than the rest of the examination days (*p* < 0.05). Additionally, at PP1 and PP2, the widest cross-sectional diameter of caruncles in ewes giving birth to twins was significantly higher than in ewes giving birth to singletons (*p* < 0.05). However, at PP3 and PP4, the widest cross-sectional diameter of caruncles in ewes giving birth to twins tended to be higher than in ewes giving birth to singletons (*p* = 0.05). In B-mod ultrasonography, the caruncles had an exact shape at PP1 but blurred and dispersed at PP7 and PP14 (Figure 2).

**Table 1:** The time-dependent differences in the evaluated parameters of the caruncles in ewes giving birth to both singletons and twins.

	PP1	PP2	PP3	PP4	PP5	PP6	PP7	PP14	*P*
Diameter									
Singleton (*n* = 9)	1.67 ± 0.09	1.50 ± 0.08	1.43 ± 0.07	1.34 ± 0.05	1.42 ± 0.07	1.36 ± 0.07	1.28 ± 0.10	1.05 ± 0.11	<0.001
Twin (*n* = 4)	2.09 ± 1.66	1.95 ± 0.16	1.81 ± 0.22	1.65 ± 0.19	1.51 ± 0.14	1.44 ± 0.14	1.25 ± 0.09	0.95 ± 0.14	<0.01
*P*	<0.05	<0.05	=0.05	=0.05	ns	ns	ns	ns	ns
CPP%									
Singleton (*n* = 9)	2.78 ± 1.05	2.67 ± 1.05	1.36 ± 0.51	1.20 ± 0.73	2.12 ± 1.30	2.01 ± 0.82	0.48 ± 0.23	0.35 ± 0.20	<0.01
Twin (*n* = 4)	7.43 ± 4.83	1.92 ± 1.00	2.73 ± 1.98	1.15 ± 0.49	4.26 ± 1.82	1.96 ± 1.45	0.45 ± 0.29	0.33 ± 0.23	<0.01
*P*	ns	ns	ns	ns	ns	ns	ns	ns	ns
Cs (m/sn)									
Singleton (*n* = 9)	2.29 ± 0.10	2.26 ± 0.11	2.55 ± 0.18	2.33 ± 0.18	2.06 ± 0.10	1.97 ± 0.15	2.04 ± 0.19	1.41 ± 0.12	<0.05
Twin (*n* = 4)	2.28 ± 0.14	2.52 ± 0.12	2.27 ± 0.15	2.61 ± 0.31	2.31 ± 0.13	2.58 ± 0.04	2.49 ± 0.21	1.89 ± 0.10	<0.05
*P*	ns	ns	ns	ns	ns	ns	ns	ns	ns
EkPA									
Singleton (*n* = 9)	16.71 ± 1.62	17.79 ± 2.23	19.74 ± 2.73	18.87 ± 3.77	14.37 ± 0.95	14.09 ± 2.86	13.82 ± 2.54	9.29 ± 1.38	<0.05
Twin (*n* = 4)	15.81 ± 2.29	19.32 ± 2.07	18.01 ± 1.91	21.19 ± 5.46	14.91 ± 2.62	20.76 ± 0.88	20.58 ± 3.18	13.24 ± 0.35	<0.05
*P*	ns	ns	ns	ns	ns	ns	ns	ns	ns

*P: Significance, ns: P < 0.05.*

**Figure 3:**
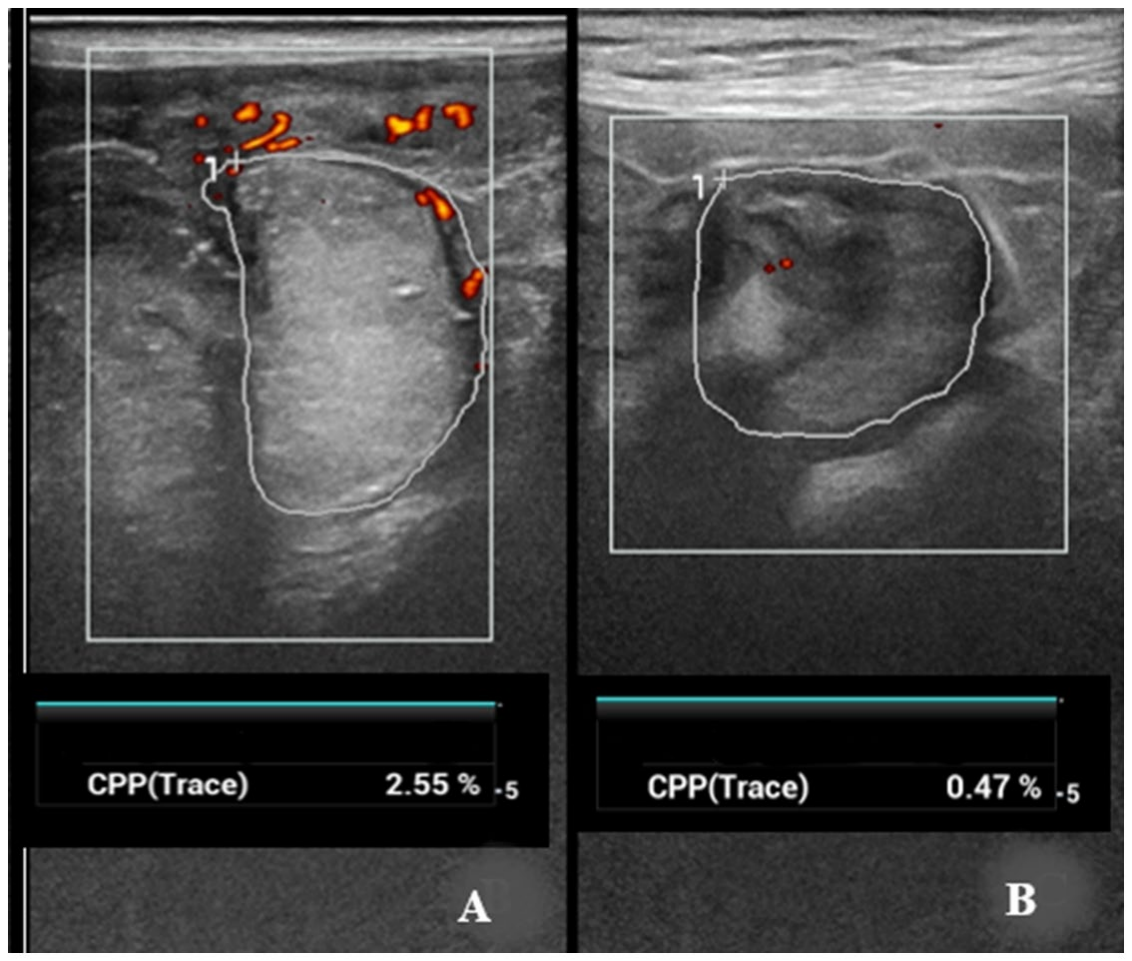
Power Doppler ultrasonographic evaluation and quantification of the vascularization on the uterine caruncles. **(A)** Quantification of colored pixels and moderate vascularization on a caruncle at PP6. **(B)** Low vascularization on a caruncle and decrease in CPP at PP14.

As a result of the power Doppler examination, CPP% were measured in uterine caruncles (Figure 3). The time-dependent differences in the CPP% were statistically significant (*p* < 0.01, respectively) in ewes giving birth to both singletons and twins (Table 1). Values of CPP at PP1 were significantly higher than at PP7 and PP14 (*p* < 0.05).

**Figure 4:**
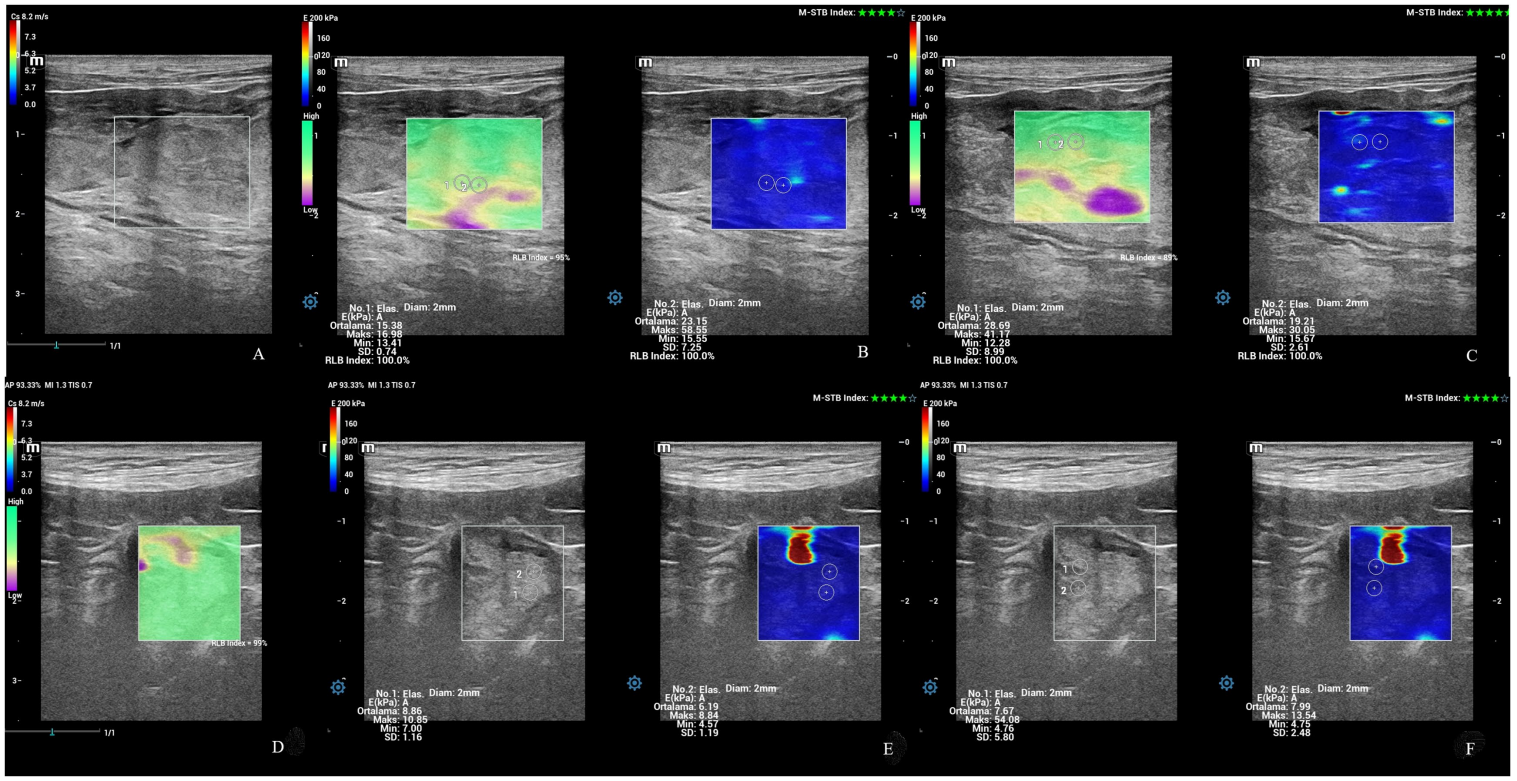
Shear wave elastography of caruncles. **(A)** Caruncule in a square ROI with a concave shape at PP1. **(B,C)** Qualitative map of shear wave elastography and quantitative evaluation of a caruncle with total 4 ROI at PP1. **(D)** An image of shear waves with 99% RLB index. **(E,F)** Qualitative map of shear wave elastography and quantitative evaluation of a caruncle with inconsistent and blurred appearance and total 4 ROI at PP14.

The time-dependent differences in SWS (m/sn) and stiffness (EkPA) in the caruncles were statistically significant (*p* < 0.05) in ewes giving birth to both singletons and twins (Table 1). The caruncular SWS (m/sn) and stiffness (EkPa) of all ewes at PP14 were significantly lower than at PP1 and PP2 (*p* < 0.05) (Figure 4).

**Figure 5:**
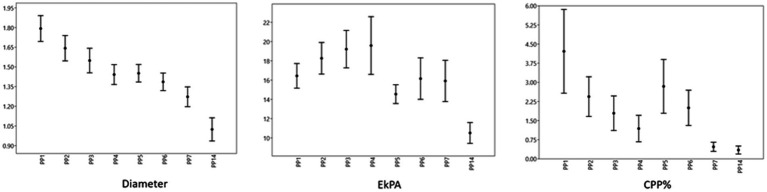
Uterine caruncle values. The point represents the median, the margins represent the percentiles (25 and 75), and the extensions of the bars represent the maximal and minimal values.

The association between the widest cross-sectional diameter, CPP%, SWS, and the stiffness of uterine caruncles was evaluated. The widest cross-sectional diameter of caruncles was not significantly associated with the SWS and stiffness of the caruncles (*p* > 0.05). At PP2 and PP5, the stiffness of the caruncles was inversely correlated with CPP% (*p* < 0.01, *p* < 0.05, respectively). The median, percentiles, maximal and minimal values of diameter, stiffness and CPP% belong to the caruncles of all ewes on the examination days are given in Figure 5.

### Cervix uteri

**Figure 6:**
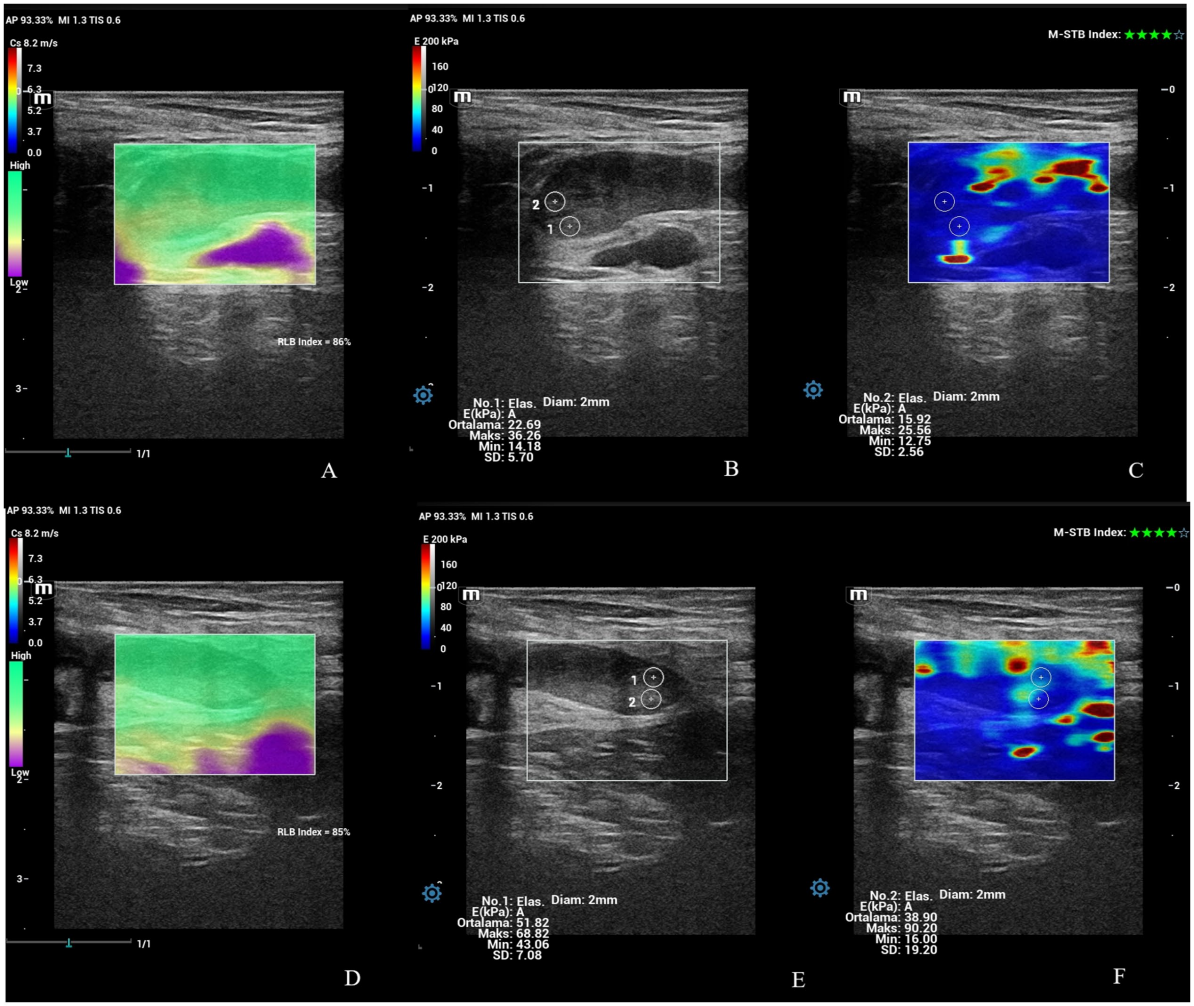
Shear wave elastography at the IOC and EOC at PP1 belonging to an ewe giving birth to twin offspring. **(A)** Shear wave image at the IOC with 86% RLB index. **(B)** Quantitative evaluation of the IOC with 2 ROI. M-STB index: 4 stars. **(C)** Qualitative map of shear wave elastography at the IOC. **(D)** Shear wave image at the EOC with 85% RLB index. **(E)** Quantitative evaluation at the EOC with 2 ROI. M-STB index: 4 stars. **(F)** Qualitative map of shear wave elastography at EOC.

**Figure 7:**
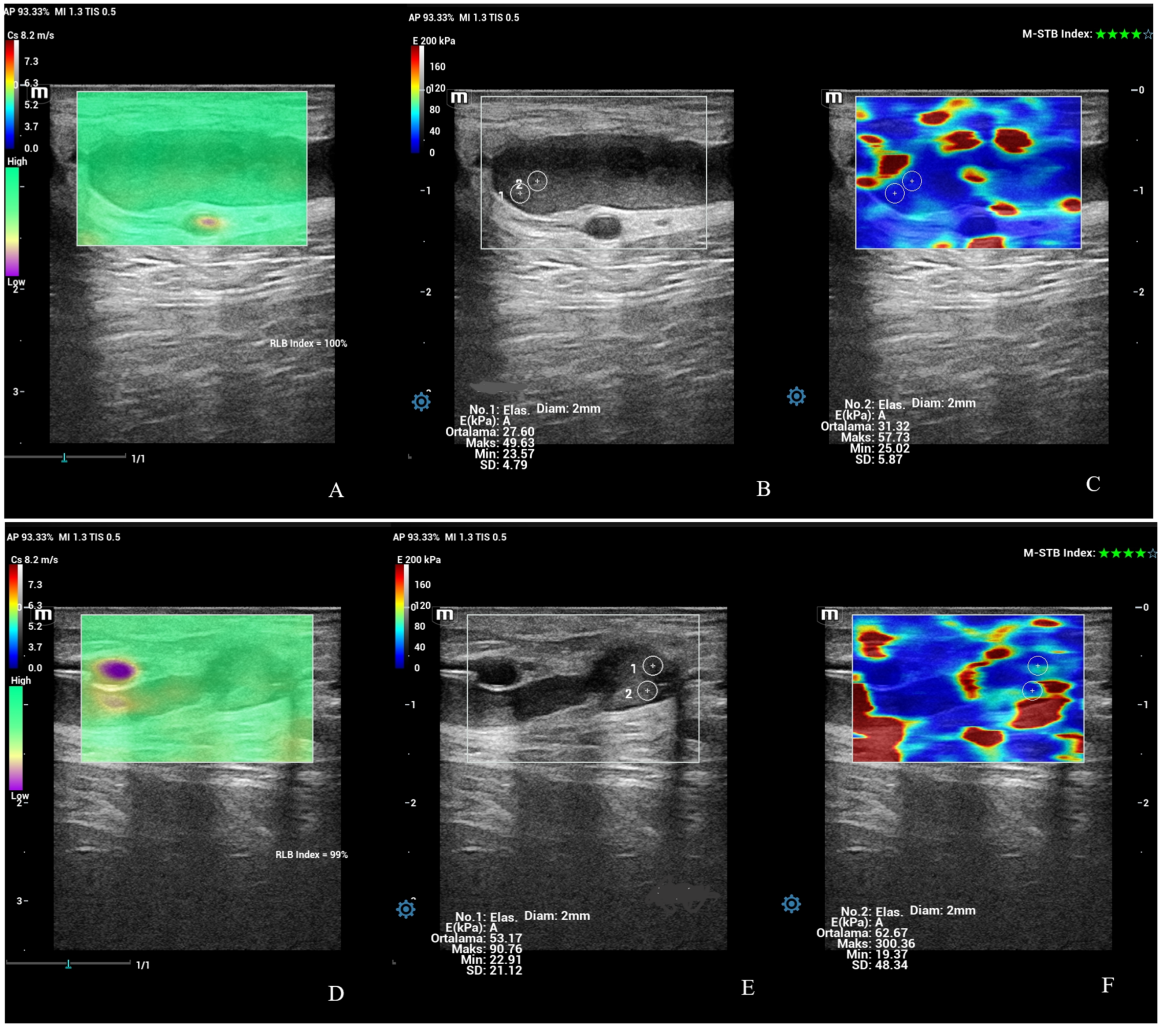
Shear wave elastography at the IOC and EOC at PP42 belonging to an ewe giving birth to singleton offspring. **(A)** Shear wave image at the IOC with 100% RLB index. **(B)** Quantitative evaluation at IOC with 2 ROI. M-STB index: 4 stars. **(C)** Qualitative map of shear wave elastography at the IOC. **(D)** Shear wave image at the EOC with 99% RLB index. **(E)** Quantitative evaluation at the EOC with 2 ROI. M-STB index: 4 stars. **(F)** Qualitative map of shear wave elastography at the EOC.

The examination of the uterine cervix included the widest diameter, SWS (Cs, m/sn), and stiffness (EkPA). The SWS and stiffness values of IOC and EOC were taken in a longitudinal view of cervix (Figures 6, 7). The time-dependent differences in the diameter of the cervix were statistically significant (*p* < 0.01) in ewes giving birth to both singletons and twins (Table 2). At PP3, the widest cross-sectional diameter of the cervix in ewes giving birth to twins was significantly higher than in ewes giving birth to singletons (*p* < 0.05). However, at PP35, the widest cross-sectional diameter of the cervix in ewes giving birth to twins tended to be higher than in ewes giving birth to singletons (*p* = 0.07). The diameter of the cervix uteri of all ewes at PP3 was significantly higher than PP14, PP21, and PP28 (*p* < 0.05). The time-dependent differences in the SWS (m/sn) and stiffness (EkPA) at the IOC were statistically significant (*p* < 0.01 and *p* = 0.001) in ewes giving birth to both singletons and twins (Table 2). The SWS at the IOC for all ewes at PP35 was significantly higher than at PP1, PP4, PP7, and PP14 (*p* < 0.05). Also, at PP35, SWS and stiffness at the IOC were significantly higher in ewes giving birth to twins compared to the ewes giving birth to singletons (*p* < 0.001). The stiffness at the IOC at PP35 for all ewes was significantly high from at PP1, PP4, PP7, and PP14 (*p* < 0.01), while it tended to be high from at PP2, PP5, and PP6 (*p* = 0.07). However, the time-dependent differences in the SWS and stiffness at the EOC were not statistically significant (*p* > 0.05). At PP3, SWS and stiffness at the EOC in ewes giving birth to singletons were significantly higher compared to the ewes giving birth to twins (*p* < 0.01). At PP21, the mean stiffness at the EOC in ewes giving birth to singletons was significantly higher compared to the ewes giving birth to twins (*p* < 0.05), however, SWS at the EOC in ewes giving birth to singletons tended to be higher than in ewes giving birth to twins (*p* = 0.08). At PP35, the stiffness at the EOC in ewes giving birth to singletons tended to be higher compared to the ewes giving birth to twins (*p* = 0.07).

**Table 2:** The time-dependent differences in the evaluated parameters of the cervix uteri in ewes giving birth to both singletons and twins.

	PP1	PP2	PP3	PP4	PP5	PP6	PP7	PP14	PP21	PP28	PP35	PP42	*P*
Diameter													
Singleton (*n* = 9)	0.65 ± 0.03	0.64 ± 0.03	0.60 ± 0.02	0.58 ± 0.03	0.64 ± 0.04	0.55 ± 0.04	0.56 ± 0.03	0.51 ± 0.03	0.52 ± 0.04	0.52 ± 0.04	0.50 ± 0.03	0.50 ± 0.03	<0.01
Twin (*n* = 4)	0.62 ± 0.09	0.71 ± 0.10	0.79 ± 0.09	0.66 ± 0.05	0.64 ± 0.06	0.67 ± 0.06	0.65 ± 0.03	0.55 ± 0.07	0.55 ± 0.07	0.55 ± 0.08	0.67 ± 0.11	0.59 ± 0.08	<0.01
*P*	ns	ns	<0.05	ns	ns	ns	ns	ns	ns	ns	0.07	ns	ns
Internal Cs (m/sn)													
Singleton (*n* = 9)	2.96 ± 0.31	2.78 ± 0.26	3.62 ± 0.5	2.7 ± 0.12	3.05 ± 0.36	3.51 ± 0.46	3.44 ± 0.32	2.93 ± 0.24	3.87 ± 0.62	3.67 ± 0.41	3.51 ± 0.24	4.00 ± 0.38	<0.01
Twin (*n* = 4)	3.44 ± 0.68	3.47 ± 0.73	3.51 ± 0.3	2.71 ± 0.61	3.48 ± 0.2	2.89 ± 0.48	3.05 ± 0.05	3.59 ± 0.23	3.53 ± 0.83	3.10 ± 0.27	6.27 ± 0.50	3.98 ± 0.51	<0.01
*P*	ns	ns	ns	ns	ns	ns	ns	ns	ns	ns	<0.001	ns	<0.05
Internal EkPA													
Singleton (*n* = 9)	30.46 ± 6.75	26.11 ± 5.16	51.92 ± 15.75	23.27 ± 2.19	32.57 ± 8.89	47.20 ± 11.83	41.20 ± 8.58	28.28 ± 4.56	59.45 ± 23.21	47.49 ± 10.30	39.86 ± 5.62	58.65 ± 9.75	=0.001
Twin (*n* = 4)	40.41 ± 17.21	42.07 ± 18.12	39.94 ± 6.6	29.74 ± 15.66	38.20 ± 4.42	29.75 ± 9.06	29.21 ± 0.67	39.98 ± 5.06	45.91 ± 21.47	30.91 ± 5.46	138.41 ± 28.79	54.04 ± 13.7	=0.001
*P*	ns	ns	ns	ns	ns	ns	ns	ns	ns	ns	<0.001	ns	<0.01
External Cs (m/sn)													
Singleton (*n* = 9)	3.49 ± 0.51	3.71 ± 0.27	4.57 ± 0.44	3.53 ± 0.32	5.35 ± 1.01	4.29 ± 0.63	4.93 ± 0.63	6.13 ± 0.96	4.23 ± 0.35	4.56 ± 0.34	5.22 ± 0.65	5.35 ± 0.80	ns
Twin (*n* = 4)	3.83 ± 0.36	2.99 ± 0.25	3.18 ± 0.10	4.79 ± 2.10	2.99 ± 0.44	3.40 ± 0.56	6.04 ± 3.01	4.13 ± 0.41	3.17 ± 0.22	3.52 ± 0.45	3.47 ± 0.26	3.62 ± 0.52	ns
*P*	ns	ns	<0.01	ns	ns	ns	ns	ns	=0.08	ns	ns	ns	<0.05
External EkPA													
Singleton (*n* = 9)	49.47 ± 18.47	45.77 ± 6.60	75.21 ± 16.59	41.76 ± 8.02	118.40 ± 44.51	71.15 ± 24.46	88.67 ± 23.41	146.20 ± 44.32	61.75 ± 12.20	69.13 ± 8.82	103.93 ± 29.40	119.07 ± 34.23	ns
Twin (*n* = 4)	47.52 ± 11.14	29.64 ± 3.28	31.55 ± 2.38	118.64 ± 101.59	29.77 ± 8.56	39.94 ± 13.57	198.50 ± 163.54	55.64 ± 11.49	31.83 ± 4.02	40.75 ± 9.97	38.20 ± 5.23	45.08 ± 12.15	ns
*P*	ns	ns	<0.01	ns	ns	ns	ns	ns	<0.05	ns	=0.07	ns	ns

*P: Significance, ns: P < 0.05.*

**Figure 8:**
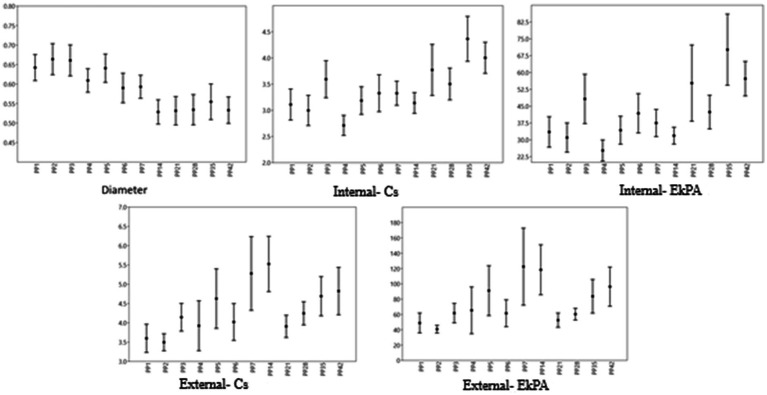
Cervix values. The point represents the median, the margins represent the percentiles (25 and 75), and the extensions of the bars represent the maximal and minimal values.

The association between the widest diameter, SWS, and stiffness of the uterine cervix was evaluated. At PP6 and PP21, SWS at the IOC was inversely associated with the diameter of the cervix (*p* < 0.01 and *p* < 0.05, respectively). However, at PP2 and PP4, SWS at the IOC tended to be inversely associated with the diameter of the cervix (*p* = 0.05 and *p* = 0.06, respectively). At PP4 and PP6, the stiffness at the IOC was inversely associated with the diameter of the cervix (*p* < 0.01 and *p* = 0.01, respectively). The widest diameter of the cervix was not significantly associated with both SWS and stiffness at the EOC in all examination times (*p* > 0.05). The median, percentiles, maximal and minimal values of diameter and elastography measurements of the cervix (IOC, EOC) of all ewes on the examination days are given in Figure 8.

### Vulva

**Figure 9:**
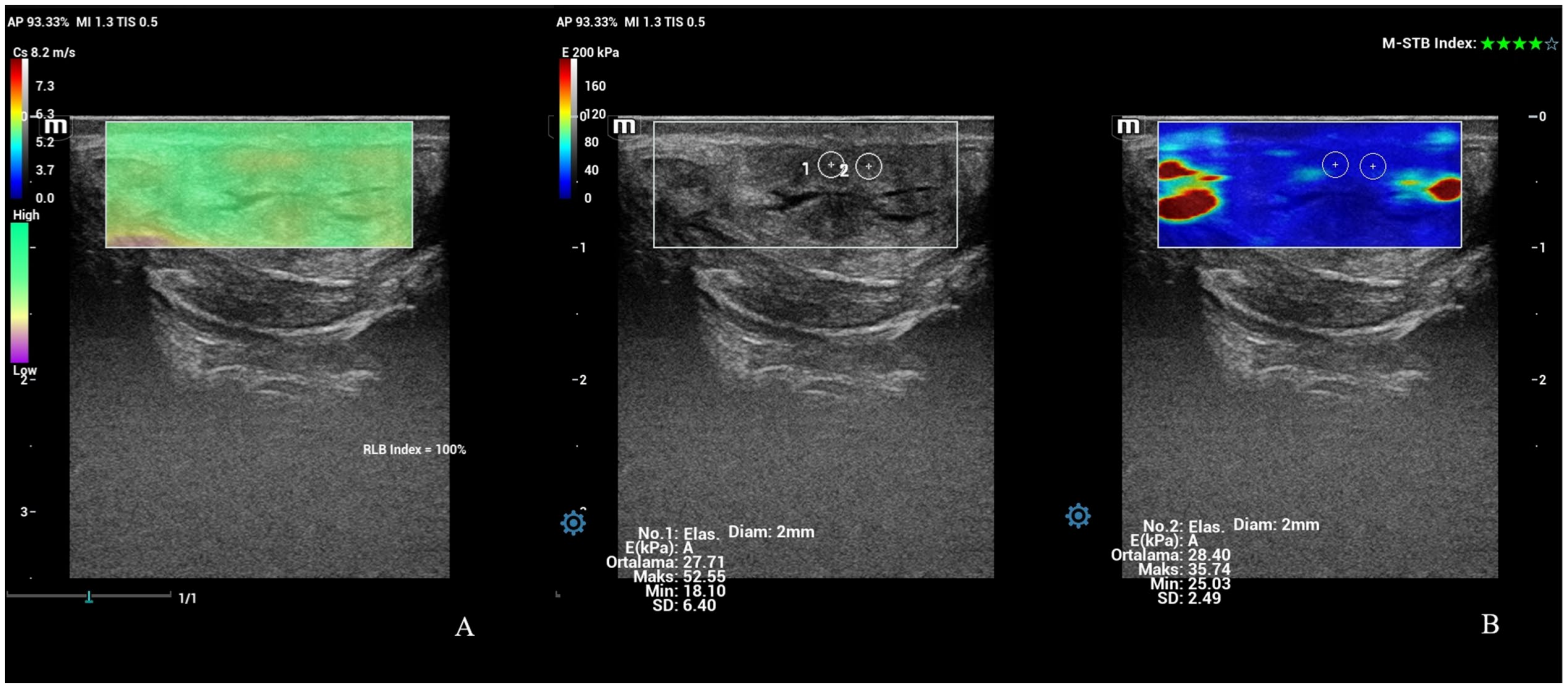
Shear wave elastography of vulva at PP6. **(A)** An image of shear waves with 100% RLB index. **(B)** Qualitative map of shear wave elastography and quantitative evaluation of a vulvar labium. M-STB index: 4 stars.

Examining the vulva, the mean thickness of both vulvar lips, SWS (Cs, m/sn), and stiffness (EkPA) (Figure 9) were evaluated. The time-dependent differences in the mean thickness of both vulvar lips were statistically significant (*p* < 0.001) in ewes giving birth to both singletons and twins (Table 3). At PP42, the mean thickness of both vulvar lips in ewes giving birth to twins was significantly higher than in ewes giving birth to singletons (*p* < 0.05). The thickness of the vulva for all ewes at PP1 was significantly high from at PP2, PP3 (*p* < 0.01) and from the other examination days (*p* < 0.001).

**Table 3:** The time-dependent differences in the evaluated parameters of the vulva in ewes giving birth to both singletons and twins.

	PP1	PP2	PP3	PP4	PP5	PP6	PP7	PP14	PP21	PP28	PP35	PP42	*P*
Thickness													
Singleton (*n* = 9)	0.51 ± 0.01	0.41 ± 0.01	0.46 ± 0.01	0.44 ± 0.01	0.433 ± 0.01	0.422 ± 0.01	0.388 ± 0.01	0.382 ± 0.01	0.383 ± 0.01	0.373 ± 0.01	0.377 ± 0.01	0.344 ± 0.01	<0.001
Twin (*n* = 4)	0.52 ± 0.02	0.47 ± 0.01	0.43 ± 0.02	0.41 ± 0.02	0.438 ± 0.01	0.436 ± 0.02	0.429 ± 0.02	0.421 ± 0.02	0.409 ± 0.02	0.405 ± 0.02	0.386 ± 0.006	0.410 ± 0.01	<0.001
*P*	ns	ns	ns	ns	ns	ns	ns	ns	ns	ns	ns	<0.05	ns
Cs (m/sn)													
Singleton (*n* = 9)	2.91 ± 0.14	3.10 ± 0.14	3.24 ± 0.17	3.14 ± 0.13	3.09 ± 0.14	3.06 ± 0.07	3 ± 0.09	3.07 ± 0.08	3.32 ± 0.11	3.28 ± 0.09	3.35 ± 0.13	3.56 ± 0.15	<0.001
Twin (*n* = 4)	2.39 ± 0.20	2.64 ± 0.10	2.89 ± 0.01	3.29 ± 0.16	2.87 ± 0.05	2.79 ± 0.04	2.90 ± 0.11	2.86 ± 0.14	3.05 ± 0.07	3.15 ± 0.15	3.28 ± 0.16	3.54 ± 0.09	<0.001
*P*	ns	ns	ns	ns	ns	<0.05	ns	ns	ns	ns	ns	ns	ns
EkPA													
Singleton (*n* = 9)	27.22 ± 2.88	29.84 ± 2.93	31.06 ± 2.78	30.15 ± 1.85	27.76 ± 1.38	30.52 ± 2.09	29.7 ± 2.15	29.3 ± 1.87	32.84 ± 1.74	35.2 ± 3	34.82 ± 2.66	44.14 ± 3.56	<0.001
Twin (*n* = 4)	18.53 ± 3.12	21.7 ± 1.67	27.03 ± 2.11	33.82 ± 3.52	29.73 ± 2.95	24.74 ± 0.51	25.87 ± 2.06	25.25 ± 2.47	28.2 ± 2.07	30.11 ± 4.21	32.39 ± 3.63	38.23 ± 4.95	<0.001
*P*	ns	ns	ns	ns	ns	ns	ns	ns	ns	ns	ns	ns	ns

*P: Significance, ns: P < 0.05.*

The time-dependent differences in the SWS (m/sn) and stiffness (EkPA) in the vulva were statistically significant (*p* < 0.001) in ewes giving birth to both singletons and twins (Table 3). At PP6, the SWS in ewes giving birth to singletons was significantly higher than in ewes giving birth to twins (*p* < 0.05). While the vulvar SWS of all ewes at PP1 tended to be low from at PP2, PP3, PP5, and PP7 (*p* = 0.07), it was significantly low from the rest of the examination days (*p* < 0.01). The vulvar stiffness of all ewes at PP1 was significantly low from at PP4, PP5 and PP14 (*p* < 0.05) and at PP21, PP28, PP35, PP42 (*p* < 0.01).

**Figure 10:**
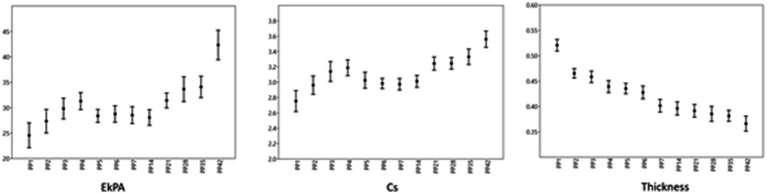
Vulva values. The point represents the median, the margins represent the percentiles (25 and 75), and the extensions of the bars represent the maximal and minimal values.

The association between the thickness, SWS, and stiffness of the vulva were evaluated. At PP35, the thickness of the vulva was significantly associated with the SWS (*p* < 0.05), while the thickness of the vulva tended to be related to the SWS at PP5 (*p* = 0.06). The stiffness of the vulva was not significantly correlated with the thickness of the vulva for all examination times (*p* > 0.05). The median, percentiles, maximal and minimal values of thickness and elastography measurements of the vulva of all ewes on the examination days are given in Figure 10.

## Discussion

The genital tract returns to the pregravid state after many progressive changes in the postpartum period. Non-invasive imaging techniques such as B-mode ultrasonography, Doppler ultrasonography, and elastography were preferred to evaluate the genital system during the postpartum period in ewes (1, 15, 18). Although there are many studies on the evaluation of uterine involution by using B-mod (6) and Doppler ultrasonography (14, 15), there are limited elastography studies on the postpartum term in ewes (1). In this study, the changes in caruncles, cervix uteri, and vulva were investigated by using non-invasive imaging techniques (B-mod ultrasonography, power Doppler ultrasonography, and shear wave elastography). To our knowledge, this is the first study to evaluate the elasticity of the caruncles, cervix uteri, and vulva during the postpartum period in ewes.

The size of the caruncles had a progressive, significant reduction afterward in the degeneration and necrosis on their surface, especially during the first week after lambing (7, 18). During the regression process, caruncles change their appearance from concave to convex imaged the caruncles with inconsistent and blurred structures at the longitudinal section taken on the fourth day postpartum during a transcutaneous examination at the inguinal area (18). In line with Ioannidi et al. (18), caruncles were concave until the fourth day of the postpartum period. Most authors have reported that imaging of caruncles by using B-mod ultrasonography after the 10th day postpartum was not possible and rare (7, 19). Differentiating the caruncles from uterine endometrial folds is usually tricky (7). Kähn (20, 21) mentioned that caruncles were visible during the second week after lambing despite their decreased size. Ababneh and Degefa (19) imaged the caruncles subsequently to the 13th day postpartum. Similar to the previous reports (19–21), there was a reduction in the size of the caruncles during the first 14 days of postpartum in both ewes giving birth to singleton offspring and twin offspring in the present study. Also, the shape of the caruncles was inconsistent, and their appearance was blurred on days PP7 and PP14 in this study. In line with the researchers (7, 19), caruncles were not visible after the evaluation point at PP14. Fernandes et al. (22) reported that the sex of newborns and single or twin partitions did not affect the placental expulsion time and cotyledons diameter (*p* > 0.05). Similarly, placental expulsion was completed within 9 h after delivery in all ewes in this study. Enginler et al. (23) indicated that Saanen’s gestation type (single and twin) and parity (primiparous and pluriparous) had no significant effect on caruncular diameter in the involution period except on day 13. However in this study, caruncular diameters of the ewes with twin parturition were significantly higher in the first 2 days of postpartum compared to the singleton parturitions.

O’Shea and Wright (11) evaluated the involution and regeneration of the endometrium following parturition in the ewes by light-and electron microscopy. Eight days after parturition, degeneration of the tissues of the placentomas was more marked, and caruncles appeared weakly vascularized. Fifteen days after parturition, the dead placental tissue had separated from the caruncles (11). Fernandes et al. (22) reported that necrotic plaques are released in caruncular involution in the second week of the postpartum period in ewes, and re-epithelialization of the caruncular region occurs during the third or fourth postpartum week. Henao-Gonzalez et al. (24) investigated the ultrasonographic screening of dairy cows with normal uterine involution, and they stated that caruncular vascular supply decreases throughout the involution process. In harmony with the previous reports, the caruncular diameter and vascularization (CPP%) of the caruncles were reduced from PP1 to PP14, regardless of the number of offspring in this study (*p* < 0.001 and *p* < 0.01, respectively). During the involution, CPP was not different between the single or twin parturition (*p* > 0.05). It was thought that the reduction of the caruncular vascularization on the postpartum period occurred due to the degeneration of the caruncular tissues in ewes.

Elastography is a non-invasive imaging technique used to evaluate tissue stiffness. Alan et al. (25) assessed the placental stiffness in pregnant women with fetal anomalies using ARFI elastography. They indicated that placental stiffness was significantly higher in pregnancies with fetal anomalies compared to healthy individuals. In addition, Alan et al. (25) did not observe a correlation between placental thickness and elasticity parameters. Da Silva et al. (17) assessed the maternal and fetal structures in pregnant ewes by using ARFI elastography. They indicated that the shear wave velocity of the placentomes remained constant through gestation in ewes. Although in this study the evaluation term was different from the previous reports, the caruncular SWS (m/sn) and stiffness (EkPa) of all ewes at PP14 were significantly lower than from at PP1 and PP2 (*p* < 0.05). The higher SWS and stiffness values of the caruncles obtained on day PP1 and PP2 could be related to the caruncular necrosis while the lower values obtained on day PP14 could be related with the release of the necrotic tissues and the onset of the re-epithelialization process (necrotic tissue; stiffer, re-epithelialization tissue; softer). In line with Alan et al. (25), the broadest cross-sectional diameter of the caruncles was not related to the elastography parameters (*p* > 0.05). To the authors’ knowledge, this is the first study that evaluates the caruncular stiffness in the postpartum involution process in ewes.

The cervix is composed of layers of collagen and smooth muscles. The structure of the cervix is associated with collagen hyaluronan, matricellular proteins, and proteoglycans, and they cause the cervix to mature. Following birth, the cervix rapidly recovers (26). The process of involution in cows was assessed by the reduced diameter of the cervix at postpartum (27). Harkness and Harkness (28) stated that the circumference of the cervix in rats falls in 8 h to about one-third of that at parturition. Subsequently, there is a slower fall until the eighth day, when the value is about an eighth of that at parturition (28). The diameter of the cervix in cows is accepted as a valuable indicator of the involution process (29). They reported that involution of the cervix in cows was affected by parity and type of postpartum discharge. The diameter of the cervix during 2 to 6 weeks postpartum regressed both in multiparous and primiparous cows with normal or abnormal discharge (29). Usmani et al. (30) observed slower involution of the cervix and uterus in buffaloes with subclinical bacterial uterus infection. Although the cervix’s involution rate is affected by the disease, it has been reported that the diameter of the cervix decreases by half when involution is completed in both healthy buffalos and those with uterine infections (30). Miettinen (31) indicated that the time duration for the involution of the cervix (approximately 30 days) was quite similar in primiparous and multiparous (3. parity) cows, and the cervix diameter looked alike at 3 weeks after parturition in primiparous and multiparous (3. parity) cows. Unlike the researchers (29, 30) in the present study, uterine infection was absent during the postpartum period in ewes, which were all multiparous. In this study, the diameter of the cervix uteri of all ewes at PP3 was significantly higher than PP14, PP21, and PP28 (*p* < 0.05). Only at PP3, the diameter of cervix in twin parturitions was higher than the ewes giving birth to singleton offspring.

The usefulness of elastography is not only valuable in obstetrics but may also play a significant role in gynecological conditions. In humans, SWS at the cervix was evaluated in healthy pregnant women (32), hysterectomy specimens of premenopausal women (33), pregnant women with pathological cervix (34) and differentiation of the benign from the malignant cervical lesions (35). In rats, the elastic and viscous behavior of the cervix was evaluated by a hydroxyproline assay, which measures collagen content (36). Mariano et al. (1) assessed the stiffness of the uterine wall parenchyma in healthy ewes during postpartum 30 days. They found that the mean shear velocity values of the uterine wall in quantitative elastography did not differ. Peralta et al. (16) investigated cervical stiffness by using shear wave elastography in pregnant ewes, which are induced with dexamethasone for cervical ripening. They observed a significant decrease in SWS in the first 4–8 h after dexamethasone compared to controls, which was thought to be associated with cervical ripening induced by dexamethasone. Studies have described elastography with significant results in veterinary research, but this is the first study to evaluate cervical stiffness during postpartum 42 days in Kivircik ewes. This study evaluated SWS and stiffness in a longitudinal view of the cervical canal on the IOC and EOC during the postpartum period. Regardless of the offspring number, the SWS and stiffness at the IOC were significantly higher at PP35 compared to the evaluation points at PP1-PP4-PP7-PP14. Liu et al. (2) reported that uterine involution was completed by day 30 postpartum in ewes with singleton parturitions, by day 35 postpartum with twin parturitions, and by day 40 postpartum with triplet parturitions. Tanaka et al. (37) measured the shear wave velocity before, immediately after, and 1 and 2 h after placental delivery to determine the cervical stiffness in women with vaginal delivery. They did not observe a significant alteration in the stiffness of the cervix over time. This may be due to the fact that the study (37) was conducted in a limited time that did not fully cover the involution period of the uterus. Although the evaluation time scale in the present study differs from the report by Tanaka et al. (37), time-dependent differences in the elasticity parameters belonging to the EOC were nonsignificant while they were significant at the IOC. The lower SWS and stiffness values at the IOC obtained on PP1, PP4, PP7 and PP14 compared to PP35 could be related to the involution process of the cervix and the change in the composition of it (ripened; soft, involuted; stiff). Although the broadest cross-sectional diameter of the cervix was significantly related to the SWS value of the IOC at PP6 and PP21, there was no relation between the diameter of the cervix and elasticity parameters of the EOC for all evaluated times. In this study, the lack of time-dependent change in SWS and stiffness values in EOC may be due to the fact that the changes in the compositions and involutions of EOC and IOC are different and these changes are more dramatic in IOC. We believe this study provides important and novel information about the objective quantification of cervical stiffness (IOC and EOC) during the postpartum period.

The vulva is the external genital organ in females, ranging from 10 cm to 12 cm in cows, while for does and ewes, it is a quarter of the length (38). During parturition, the cervix, vagina, and vulva are the physical and anatomic barriers (39). At the parturition, some externally visible changes occur in animals. The most important external changes of approaching parturition are seen in the udder, vulva, and pelvic ligaments and, to some extent, in the behavior (40). The vulva becomes progressively edematous, flaccid, and enlarged (2 to 5 times the average size) as parturition approaches in most domestic animals (41). Saut et al. (42) investigated vulvar edema in dairy cows for 43 postpartum days. The severity of vulvar edema disappeared after the 3rd postpartum day, and a significant decrease in vulvar edema was observed until the 43rd postpartum day (42). The thickness of the vulva for all ewes at PP1 was significantly high from at PP2, PP3 (*p* < 0.01) and from the other examination days (*p* < 0.001). The decrease in the thickness of the vulva is due to the reduction of vulvar edema in the postpartum period. The SWS and stiffness values of the vulvar lips at PP1 were significantly lower compared to at PP14, PP21, PP28, PP35, and PP42. This may be due to the softness of the vulvar lips as a result of the edema at PP1. To the best of our knowledge, this is the first study in ewes to evaluate the elasticity of the vulva in the postpartum period.

## Conclusion

Recent studies have reported ultrasonography (B-mode and Doppler) as a valuable tool for evaluating the involution process in ewes. In our research, it was possible to describe the changes throughout the postpartum period and assess the involution of uterine cervix, caruncles, and vulvar labia and tissue stiffness during postpartum involution by B mode ultrasonography, power Doppler ultrasonography, and shear wave elastography with significant results. We provided valuable information to elucidate the differences in the involution process concerning the offspring number during the postpartum period in Kivircik ewes. Shear wave elastography may be a valuable method for objectively quantifying the stiffness of the gynecological tissue and organs.

## Data Availability

The original contributions presented in the study are included in the article/supplementary material, further inquiries can be directed to the corresponding author.
